# Recombinant Decoy Exhibits Broad Protection against Omicron and Resistance Potential to Future Variants

**DOI:** 10.3390/ph15081002

**Published:** 2022-08-15

**Authors:** Haoneng Tang, Yong Ke, Lei Wang, Mingyuan Wu, Tao Sun, Jianwei Zhu

**Affiliations:** 1 Engineering Research Center of Cell and Therapeutic Antibody, Ministry of Education of China, School of Pharmacy, Shanghai Jiao Tong University, Shanghai 200240, China; 2 School of Agriculture and Biology, Shanghai Jiao Tong University, Shanghai 200240, China; 3 Shanghai Municipal Veterinary Key Laboratory, Shanghai 200240, China; 4 Jecho Biopharmaceuticals Co., Ltd., Tianjin 300467, China; 5 Jecho Laboratories, Inc., Frederick, MD 21704, USA; 6 Jecho Institute, Co., Ltd., Shanghai 200240, China

**Keywords:** SARS-CoV-2, future variants, recombinant receptor decoy therapeutic, neutralizing antibody, cocktails, resistant profile

## Abstract

The Omicron variant has swept through most countries and become a dominant circulating strain, replacing the Delta variant. The evolutionary history of severe acute respiratory syndrome coronavirus 2 (SARS-CoV-2) suggests that the onset of another variant (possibly another variant of concern (VOC) is inevitable. Therefore, the development of therapeutics that enable treatments for all Omicron-included VOCs/variants of interest (VOIs) and future variants is desired. Recently, the recombinant receptor decoy therapeutic angiotensin-converting enzyme 2 (ACE2)-Fc has exhibited good safety in a phase 1 clinical trial; therefore, its variant-resistant profile needs to be understood. Here, we conducted a comprehensive evaluation of its neutralization breadth against the Omicron variant and other VOCs/VOIs. Furthermore, to evaluate its resistance to future variants, we investigated its ability to neutralize various single-residue mutated variants. Next, we demonstrated its resistance to evasion via an experiment that rapidly and effectively stimulates virus evolution with a replication-competent virus model. In addition, we evaluated its efficacy for cocktail therapy. The combination of ACE2-Fc and neutralizing antibodies showed both efficacy and breadth in the simulation experiment. The underlying mechanism was revealed to be a synergistic effect in the cocktails. Collectively, this study deepens the understanding of the resistance profile of recombinant receptor decoy therapeutics and highlights the potential value of ACE2-Fc and neutralizing antibody cocktails in the subsequent anti-SARS-CoV-2 campaign. Furthermore, we also provide an effective method to study the resistance profile of antiviral agents and rapidly screen for potential cocktails to combat future variants.

## 1. Introduction

Omicron variant has overtaken the Delta variant and spread rapidly worldwide, thereby becoming the most dominant variant circulating in most countries (e.g., USA, United Kingdom, Germany, and Denmark) [1]. In March 2022, the highly transmissible Omicron was detected in mainland China, plunging tens of millions of people into lockdown [2]. Despite the increased transmissibility of Omicron, some countries are easing their restriction and surveillance efforts, partly because Omicron is less likely to cause severe disease and death than previous severe acute respiratory syndrome coronavirus 2 (SARS-CoV-2) variants. The lower severity of the Omicron variant may be due to an intrinsic property in which it shows a preference for infecting the upper respiratory tract rather than lung tissue [3]. However, elderly patients and those with underlying conditions continue to be at a risk of developing severe disease.

The history of SARS-CoV-2 evolution suggests that a new variant will be detected every few months, and there is speculation that the virus could evolve into a strain that induces milder symptoms. However, the evolutionary pathway of SARS-CoV-2 remains unclear. There is no guarantee that the next dominant variant will evolve from the ‘mild’ Omicron. Therefore, it is possible that future variants may emerge from a separate virulent lineage with sufficient immune evasion to break through the herd immunity built up through vaccination and prior infection.

Antibodies and antibody-like products have become the most promising therapies for Coronavirus Disease 2019 (COVID-19), as demonstrated by the Emergency Use Authorization (EUA) antibodies. However, this perception is wavering, as the efficacy of these authorized antibody therapeutics was abolished by Omicron (e.g., casirivimab and imdevimab cocktail [4,5,6], and sotrovimab [7]). This provides us with a sobering cognition that both combination and/or conserved epitope strategies cannot protect antibody therapeutics from escape mutations acquired by virus evolution. During natural evolution, the virus is under the intense selective pressure of the human immune system, which contains countless combinations of antibodies with conserved or non-conserved epitopes. Thus, the virus evolves toward evading antibody-based therapeutics. However, regardless of how the virus evolves, it must maintain or even enhance its receptor binding capacity to ensure infection. Therefore, receptor decoy therapy is, in principle, the most competent therapeutic in the context of viral evasion. Angiotensin-converting enzyme 2 (ACE2) serves as the primary receptor for SARS-CoV-2 entry into host cells [8,9,10,11]. ACE2 decoy therapy has already been studied in the early period of the COVID-19 pandemic [12,13]; however, its clinical research progress has not been emphasized and enthusiastic enough as compared to that of antibody therapeutics. Neutralizing therapeutic antibodies were fast-tracked into adaptive phase 1/2/3 clinical trials and authorized marketing approval in only 9–10 months from the initial discovery of these antibodies [14,15,16,17,18]. Recently, on 14 March 2022, a recombinant human ACE2 fusion protein with IgG1 Fc at the C-terminal (ACE2-Fc) demonstrated good safety and tolerability in a phase 1 clinical study [19], primarily based on favorable results from a previous in-vitro and in-vivo antiviral study in March 2021 [20]. Thus, its neutralizing ability against Omicron should be investigated in a timely manner and its resistance to future SARS-CoV-2 variants should be fully understood. Furthermore, it is unclear whether ACE2-Fc can be combined with neutralizing antibodies.

Here, we generated the ACE2-Fc fusion protein by fusing the extracellular domain of the human ACE2 receptor with the Fc domain of human IgG1, and then expressing it with the ExpiCHO expression system. After protein A purification, the ACE2-Fc product was obtained and we investigated its neutralizing ability against the dominant circulating Omicron variant as well as other variants of concern/variants of interest (VOCs/VOIs). We then evaluated its broad neutralization ability against various single-residue-mutated variants. Next, we further explored its resistance to future variants using a rapidly replicating and evolving virus model that carried the spike protein of SARS-CoV-2. Finally, we evaluated the cocktail potential using neutralizing antibodies in a virus evolution simulation experiment.

## 2. Results

### 2.1. ACE2-Fc Neutralizes VOCs/VOIs including Omicron Variant

**Figure 1:**
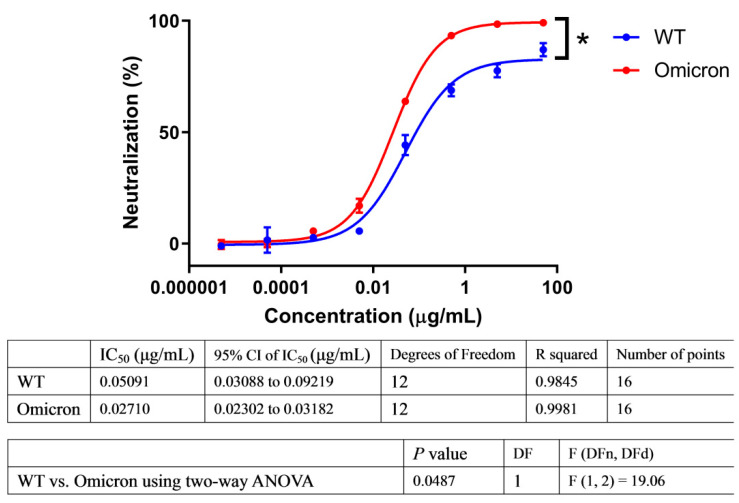
ACE2-Fc showed enhanced neutralization ability against Omicron. Lentiviral particles pseudotyped with S protein of wild-type (WT) or Omicron were used to evaluate their susceptibility to ACE2-Fc neutralization in parallel. Neutralization ratios were calculated based on luminescent units detected (mean ± SD, *n* = 2). Dose-response neutralization curves were fit to a four-parameter logistic equation by nonlinear regression analysis. Means were compared using two-way ANOVA statistical analysis with testing level (alpha) of 0.05. * *p* < 0.05.

To evaluate the neutralization ability of ACE2-Fc against SARS-CoV-2 variants, we employed a commonly used HIV-based pseudovirus carrying the spike protein of SARS-CoV-2 variants to compare its neutralizing potency against Omicron in parallel with the Wuhan-Hu-1 wild type (WT) strain. In this proof-of-principle assay, we found that ACE2-Fc efficiently neutralized the Omicron variant and showed enhanced rather than reduced neutralizing potency against the pseudovirus (Figure 1), which may be due to the fact that SARS-CoV-2 evolved to have a better engagement with the ACE2 receptor. We also evaluated and demonstrated the breadth of its inhibitory potency against all the variants of concern/variants of interest (VOCs/VOIs), including Alpha, Beta, Gamma, Delta, Eta, Iota, Kappa, Lambda, and Mu, as well as SARS-CoV-1 (Figure S1). Although these variants no longer circulate widely around the world, it is important to understand the neutralizing generality of ACE2-Fc. It is possible that a future variant may be the descendent of these lineages if they accumulate sufficient mutations to overcome Omicron.

### 2.2. ACE2-Fc Neutralizes Various Single-Residue Mutated Variants That Escape EUA Therapeutic Antibodies

**Figure 2:**
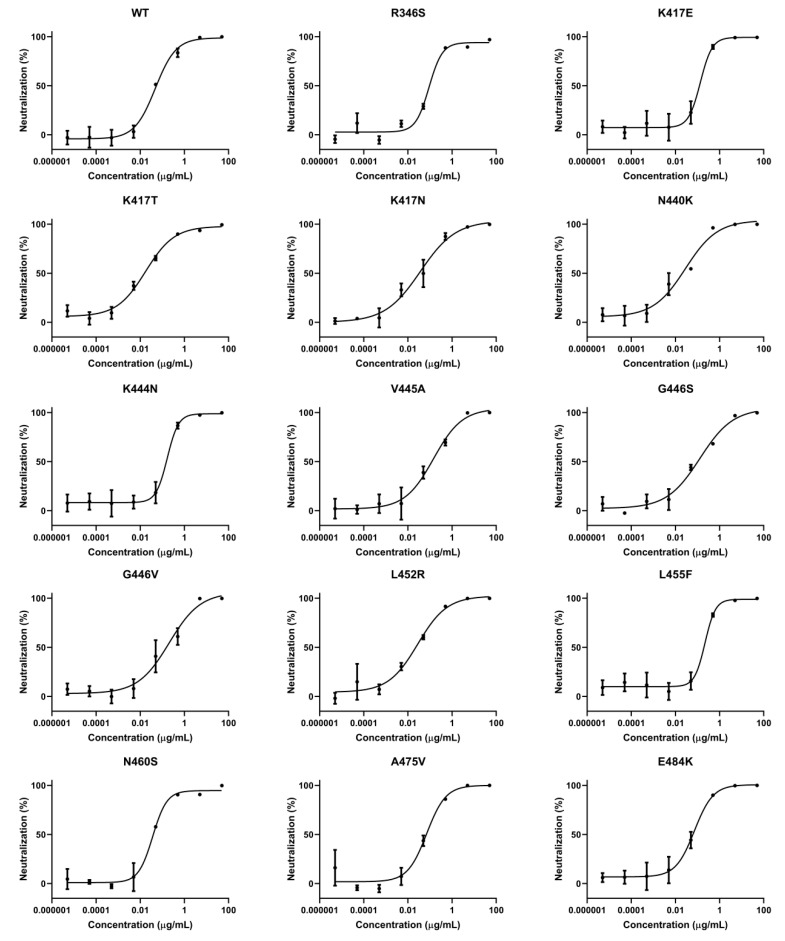

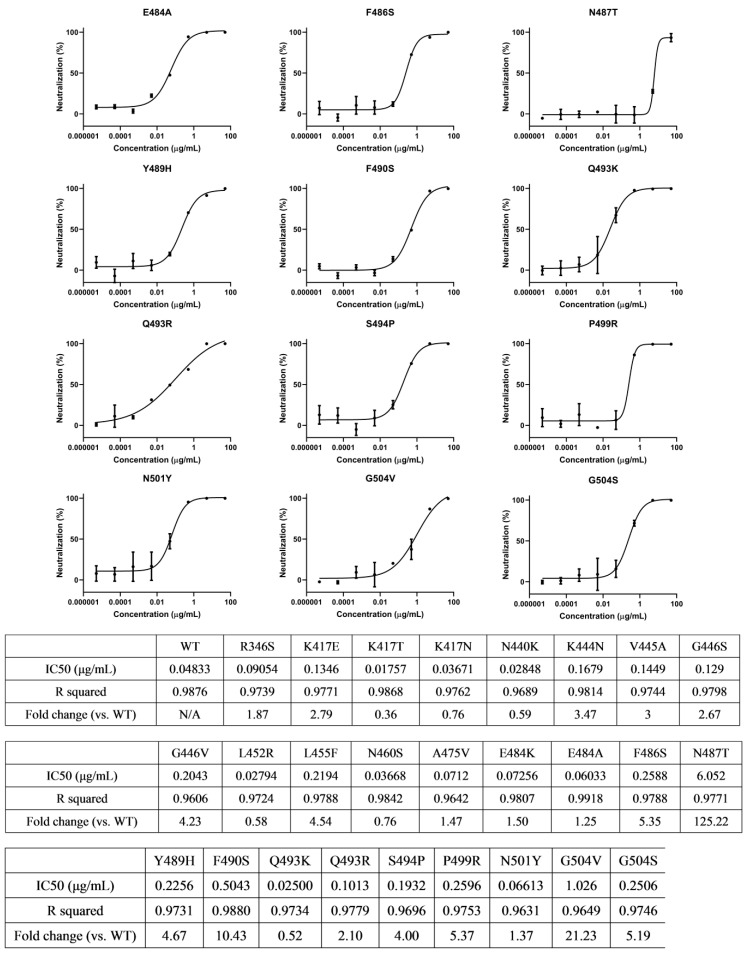
ACE2-Fc efficiently neutralized various single-residue mutated variants. The variants in these tables were found to escape antibody therapeutics either Emergency Use Authorization (EUA) authorized or in clinical trials, according to references listed in Figure S2. Neutralization ratios were calculated as described in Figure 1 (mean ± SD, *n* = 2). IC_50_ values were calculated using four-parameter nonlinear regression fitting.

We have summarized some single amino acid mutations that were selected in the lab upon the selective pressure of antibody therapeutics (either EUA-authorized or in clinical trials) [21,22,23,24,25,26,27] (Figure S2). To evaluate the neutralization ability of ACE2-Fc against these antibody-resistant variants, we conducted site-directed mutagenesis based on the sequence of the wild-type (WT) spike to construct these single-residue mutated sequences, which were then assembled into HIV-based pseudovirus variants carrying these mutated spike proteins. Subsequently, the ability of ACE2-Fc to neutralize these variants was evaluated. We found all the single-residue mutated variants were neutralized by ACE2-Fc (Figure 2). However, we also found that a mutation at residue position 487 (N487T) significantly affected ACE2-Fc neutralization by approximately 100-fold (Figure 2). Despite reduced neutralization, ACE2-Fc at a high concentration (50 μg/mL) was still able to fully neutralize the N487T variant (Figure S3B). Therefore, we demonstrated that ACE2-Fc was resistant to various single-residue mutations that may be carried by future variants.

### 2.3. ACE2-Fc Neutralizes Variants of Replication-Competent Virus Model

To evaluate the value of ACE2-Fc in combating future potential variants, we designed a viral model that mimics the evolution of SARS-CoV-2 using a replication-competent chimeric virus based on vesicular stomatitis virus (VSV) encoding the SARS-CoV-2 spike protein and green fluorescent protein (GFP). We designated this chimeric virus VSV-SARS-CoV-2-S.

This replication-competent virus model was employed in a neutralization assay to evaluate the resistance profile of ACE2-Fc against high quantities of spike mutations that may be carried by future variants. The infectious virus population was generated as described in Section 4, followed by seven passages to generate initial sequence diversity. The viral population was then incubated with serially diluted ACE2-Fc to neutralize the susceptible variants. The ACE2-Fc-virus mixture was then applied to Vero E6 cells, which supported robust VSV-SARS-CoV-2-S replication. After incubation for 72 h, cells were monitored for GFP expression. The viral supernatant containing the suboptimal concentration of ACE2-Fc showing evidence of viral replication was harvested. The clarified supernatant was incubated with serially diluted ACE2-Fc and used to infect fresh Vero E6 cells for the next passage, as previously described. This procedure was repeated 15 times (3 d/round) (Figure S4). We hypothesized that this 45-day procedure would generate a large population of variants.

**Figure 3:**
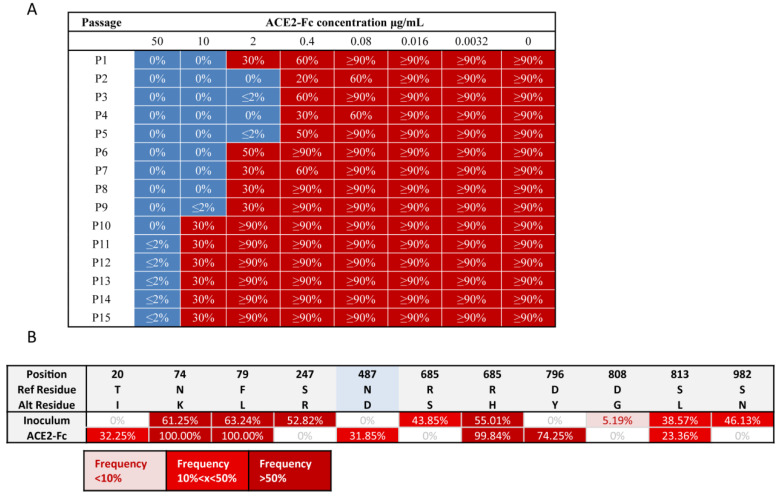
ACE2-Fc neutralized a replication-competent virus model that produced vast quantities of mutations on spike protein in the 45 d viral evolution. (**A**) Throughout this 45 d continual replication and evolution, VSV-SARS-CoV-2-S virus population failed to generate a variant that was able to escape ACE2-Fc. Replication-competent VSV-SARS-CoV-2-S virus was neutralized by serial concentrations of ACE2-Fc and then applied to Vero E6 cells. After 72 h, the cells were monitored for GFP expression and the suboptimal concentration of ACE2-Fc that permitted viral replication (>20% GFP-positive cells), was recorded. This viral supernatant was then incubated with serial diluted ACE2-Fc and used to infect fresh Vero E6 cells for next passage, as previously. This procedure was repeated 15 rounds (3 d/round). A representative record of GFP percentage in each well from one of two independent experiments is shown here. (**B**) Mutations on the spike proteins carried by the passage 15th viral population. RNA was extracted from the supernatant of passage 15th (p15) viral population and subjected to viral genome high-throughput sequencing using Illumina. Because of the error rate of Illumina sequencing, the prevalence of low frequency mutations (<5%) was extremely difficult to be accurately measured. Thus, only mutations occupying over 5% viral population were shown. RBD region was highlighted in blue. Mutation N487D within the RBD was presented at 31.85% frequency in the p15 population.

Throughout this 45 d continual replication and evolution, the VSV-SARS-CoV-2-S viral population failed to generate a variant that could escape ACE2-Fc (Figure 3A). To reveal the final mutation landscape, the passage 15th (p15) viral population was subjected to high-throughput sequencing of the viral genome using Illumina. The sequencing results reveal that the mutation N487D within the RBD region was present at a 31.85% frequency in the p15 population (Figure 3B). As previously mentioned, mutation at residue 487 reduced ACE2-Fc neutralization (Figure S3B). However, it is understandable that if the viral population evolves to reduce ACE2-Fc neutralization, they also sacrifice their ability to bind to the ACE2 receptor, which is detrimental to viral infection. Therefore, the partial viral population (31.85%) mutated into D487 may indicate a balance between reducing neutralization by ACE2-Fc and enhancing engagement with the ACE2 receptor to ensure infection. Overall, this assay result demonstrates that ACE2-Fc may be able to resist vast quantities of spike mutations to retain its neutralization ability throughout the 45 d viral evolution.

### 2.4. ACE2-Fc Is Valuable for Cocktail Therapy

**Figure 4:**
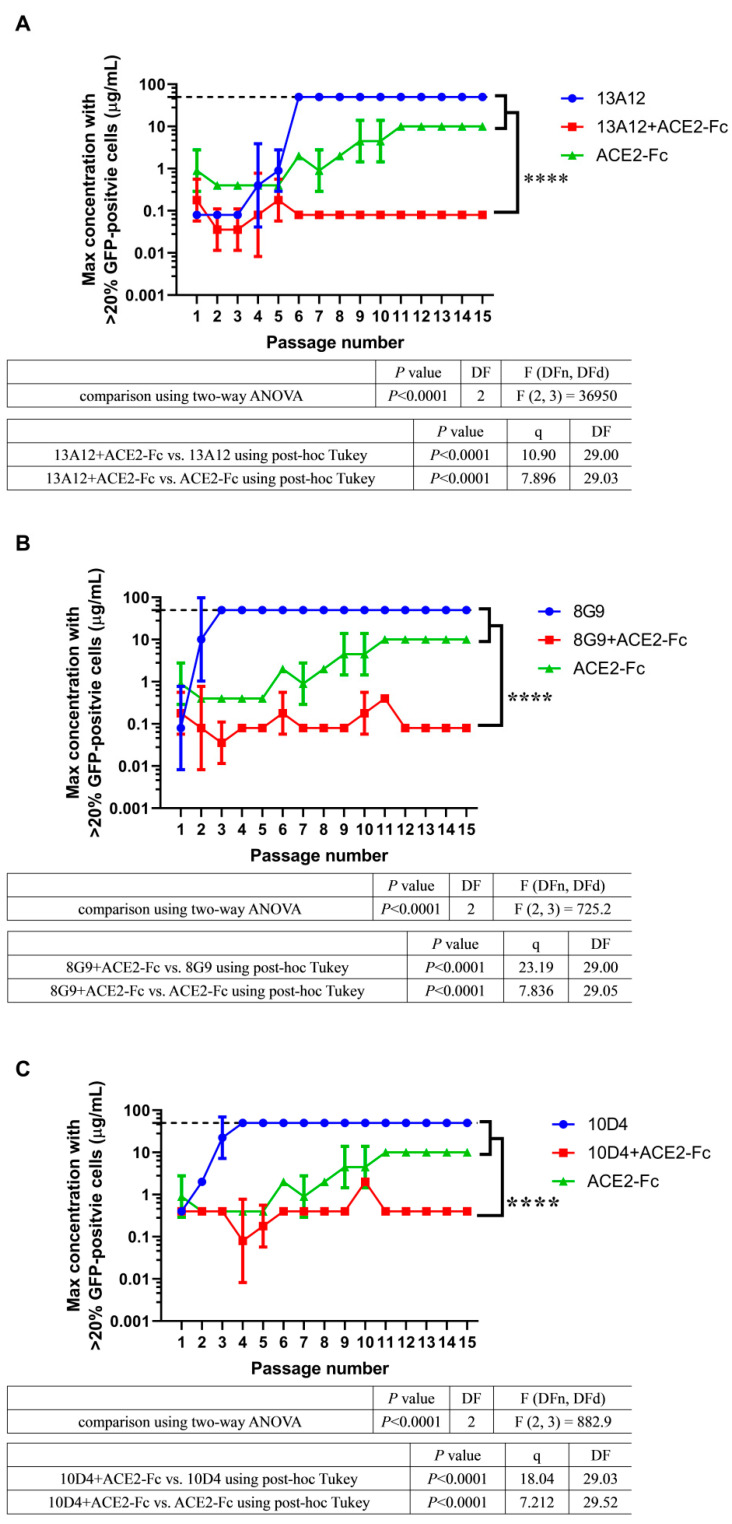
ACE2-Fc has the potential for cocktail therapy. Three cocktails, (**A**) ACE2-Fc + 13A12, (**B**) ACE2-Fc + 8G9, and (**C**) ACE2-Fc + 10D4, were evaluated in the 45 d VSV-SARS-CoV-2-S viral evolution. These cocktails achieved both efficacy and breadth throughout the evolution of the virus. The dashed line represents the concentration of 50 μg/mL. Within six passages, viruses have evolved to escape 50 μg/mL antibodies. The suboptimal concentrations that permitted viral replication from two independent experiments (*n* = 2) are shown as mean ± SD. The passage number–suboptimal concentration curve of ACE2-Fc from Figure 3A was superimposed to graphs of each cocktail. The significant differences were evaluated using two-way ANOVA statistical analysis with a post hoc correction (Tukey test) for family-wise error rate. **** *p* < 0.0001.

To evaluate whether ACE2-Fc is valuable for combining with neutralizing antibodies, we used three potent neutralizing antibodies, 13A12, 8G9, and 10D4, which originated from COVID-19 convalescent patients [28]. These three cocktails (ACE2-Fc + 13A12, ACE2-Fc + 8G9, and ACE2-Fc + 10D4) were evaluated in the 45 d VSV-SARS-CoV-2-S virus evolution. All cocktails sustained efficacy throughout the evolution of the virus, with no escape variant emerging (Figure 4). However, the neutralizing antibodies alone were escaped within six passages (Figure 4), indicating that they could not resist some spike mutations that had taken place during viral evolution.

The escaped viral population and the p15 viral population of cocktails were subjected to high-throughput sequencing. For all escaped viral populations, mutations presented at high frequencies indicated that they were selected under the selective pressure of neutralizing antibodies (Figure S5). However, these mutations were not present or were present at a low frequency in the p15 viral population of corresponding cocktails (Figure S5). In the case of 13A12, mutation K417E within the RBD region was present at 100% frequency in the escaped population, whereas this mutation accounted for only 13.61% of the p15 population in the ACE2-Fc + 13A12 cocktail (Figure S5A). Viruses that evolved in the presence of 8G9 had mutations at positions N354, D389, and E484, but these mutations did not emerge upon treatment with the ACE2-Fc + 8G9 cocktail (Figure S5B). A similar result was observed in 10D4; mutation I468T, which was selected in the escaped population, did not emerge in the cocktail treatment (Figure S5C). Together, these sequencing results suggest that the mutations that escaped the neutralizing antibodies might have been suppressed by the component ACE2-Fc in the cocktail therapies. To confirm this hypothesis, we investigated the ability of ACE2-Fc to neutralize these three escaped viral populations (13A12, 8G9, and 10D4). Escaped viral populations were harvested and neutralized by serially diluted ACE2-Fc, and then applied to Vero E6 cells to measure GFP fluorescence by flow cytometry. We found that ACE2-Fc efficiently neutralized these three viral populations (Figure S6). Overall, ACE2-Fc has the potential to be combined with neutralizing antibodies, thus achieving better efficacy in suppressing the evolving viral population. This may be due to the synergistic effect in which component ACE2-Fc suppresses the outgrowth of a neutralizing antibody-resistant viral population for another component neutralizing antibody to efficiently neutralize the rest of the viral population.

## 3. Discussion

In addition to the prevalence of VOCs [29,30,31,32,33], an increasing number of antibodies have been reported to have their neutralizing ability abolished or impaired [4,5,6,7,27,34,35,36]. Although there is an option for scientists to screen out and develop a new antibody for the new variant, this approach is costly and laborious. It is difficult to regard this approach as sustainable, as scientists need to make great efforts to combat the costless and effortless natural evolution of SARS-CoV-2. In contrast, if we developed an ACE2-Fc therapeutic as a weapon against this, then the situation would be completely different. In this study, we found that ACE2-Fc shows enhanced, rather than reduced, neutralization ability against Omicron (Figure 1). Therefore, the initial effort in drug development effortlessly derives a better efficacy as the virus has evolved to enhance its receptor engagement, standing in stark contrast to the antibodies.

Currently, antibody therapeutics are conditionally applied in clinics to treat patients with COVID-19. However, a serious problem associated with these antibodies is that they have a risk of selecting treatment-induced escape variants [22,37]. The amino acid mutations within these escape variants may seed into the viral population and be carried by future variants. We assembled 26 variants with single-residue mutations on RBD and found that ACE2-Fc can neutralize all of them. Another similar study constructed an RBD mutant library containing each single-residue mutation on RBD using deep mutational screening (DMS) technology and demonstrated that an ACE2 decoy can neutralize each of them [38].

Among the antibody-resistant single-residue mutated variants, the N487T variant was relatively refractory to ACE2-Fc neutralization. In fact, two studies have reported that the N487T RBD mutant has attenuated binding to the ACE2 receptor [21,25]. This indicates that N487 is a critical amino acid in the interaction between the spike protein and ACE2 receptor. We downloaded a high-resolution RBD–ACE2 complex structure model (PDB ID:6M17) [11] and examined the atomic interaction between N487 of RBD and ACE2. We found that two hydrogen bonds are formed between RBD and ACE2 (N487-Y83 and N487-Q24) (Figure S3A). We also used a smoothed backbone-dependent rotamer library [39] to predict the N487T mutated structure. We found that T487 completely lost its interaction with ACE2 (Figure S3A), which may explain why ACE2-Fc has attenuated neutralization potency against the N487T variant. However, the N487T variant was still fully neutralized by a high concentration of ACE2-Fc (Figure S3B). This indicates that the ACE2 decoy was more resistant to viral mutations than neutralizing antibodies, and the neutralizing ability of the latter was usually fully abolished by a single amino acid mutation [22,28,31]. To determine the structural reason for this, we calculated and marked residues on RBD within a 4 Å distance from ACE2 using Chimera (see Materials and Methods) to illustrate the ACE2-RBD interface. There was a large attachment area and large quantities of interacting residues between ACE2 and RBD (Figure S3C); therefore, mutation at one residue position may be relatively minor to affect the interaction network. Moreover, according to the sequence statistics in CoV-GLUE [40], by November 2021 there were only four virus sequences identified with the N487T mutation worldwide. This implies that this mutation may be extremely detrimental to viral fitness and is unlikely to be carried by future variants.

In order to evaluate the resistance profile of ACE2-Fc, we generated a replication-competent VSV-SARS-CoV-2-S virus model. Notably, the VSV-SARS-CoV-2-S virus replicates rapidly and to high titers (10^7^ to 10^8^ PFU/mL within 48 h) [41,42,43]. The absence of proof-reading activity in the viral polymerase results in a mutation rate of ~10^−4^ to 10^−5^/base per replication cycle [44,45,46], which is higher than that of authentic SARS-CoV-2 [47,48,49,50]. Moreover, replication of the VSV-SARS-CoV-2-S virus can be readily monitored and measured using GFP fluorescence. These features allow this virus model to mimic the vast quantities of spike mutations generated during SARS-CoV-2 evolution.

Previous studies have suggested that the wide application of antibody therapeutics can drive viral evolution in a specific direction [22,23,28]. That is, antibody therapeutics select some treatment-induced escape variants and accelerate their expansion in the viral population to become dominant. This drawback may also lead to undermining the effectiveness of other antiviral agents or vaccines. The evolutionary process of SARS-CoV-2 seems to be accelerated, as Omicron has accumulated an unprecedentedly larger number of mutations than Delta [33]. It is uncertain to what extent this acceleration should be attributed to the wide use of antibody therapeutics, but the emergence time of the Omicron variant (November 2021 [33]) coincided with the time after the marketing of antibody therapeutics worldwide (from November 2020 [51]–May 2021 [52]). This possible relevance may be an important issue for future studies. Our study shows that undetectable escape variants in the viral population could be steeply accelerated to become dominant under selective pressure of antibodies (Figure 4). Moreover, previous studies have shown even the combination of two neutralizing antibodies may be escaped by the replication-competent VSV-SARS-CoV-2-S (combination of REGN-10933 and REGN-10987 was escaped in passage seven and combination of COV2-2130 and COV2-2196 was escaped in passage five) [37]. However, for the ACE2-Fc therapeutic, we demonstrated in a 45 d virus evolution study that it did not induce escape variants (Figure 3). Therefore, the ACE2-Fc therapeutic holds the promise of significantly restricting the emergence of escape variants and is thus less likely to undermine other available therapeutics and vaccines when applied in the clinic, as compared to antibody therapeutics.

Despite the broad resistance profile of the ACE2 decoy, ACE2-Fc therapy was deemed inferior to neutralizing antibodies owing to its relatively weak neutralization ability. The IC_50_ of ACE2-Fc neutralizing activity against authentic SARS-CoV-2 is ~5 nM [53]; however, neutralizing antibodies usually achieve picomolar inhibition [28,54,55,56]. Therefore, we evaluated whether ACE2-Fc is valuable for combining with neutralizing antibodies. We found that the cocktails not only did not induce escape variants, but also inherited the efficacy of the component antibodies (Figure 4). We demonstrated that this is due to a synergistic effect in which ACE2-Fc inhibits the outgrowth of antibody-resistant variants for the potent antibody to continue to exhibit its efficacy. These combinations may be valuable for prophylaxis and treatment of COVID-19 because they would mitigate the treatment failure caused by the emergence of treatment-induced escape variants seen in single antibody treatment. Scientists are continuously struggling to develop potent neutralizing antibodies against Omicron [57,58,59,60,61,62,63,64,65,66,67,68,69,70]. It is important that combinations of these available therapeutics should be considered and explored. A crucial question that remains to be addressed is whether these combinations could be deployed as an effective countermeasure against future variants. To proactively prepare for the inevitable coming of the next variant, we propose to evaluate these Omicron-neutralizing antibodies and ACE2-Fc therapeutic combinations for their efficacy in virus evolution simulation assays. The replication-competent VSV-SARS-CoV-2-S virus model has provided a robust and readily deployable platform for us and others to carry out this future-variant resistant potential screening.

Moreover, although some accessory receptors or attachment factors (e.g., neuropilin-1 [71,72], C-type lectin CD209L/L-SIGN and the related protein CD209/DC-SIGN [73], and cellular heparan sulfate [74]) have been proposed to play a role in the entry of SARS-CoV-2 into host cells, only ACE2 is an essential entry receptor. A study using CRISPR/Cas9 genetically engineered human organoid biobanks demonstrated that the knockout of these co-receptors did not significantly affect viral replication in the organoids, whereas ACE2-deficient organoids were fully resistant to SARS-CoV-2 infection [75]. This suggests that ACE2 decoy therapy has the highest clinical value among receptor decoy therapeutics against SARS-CoV-2. In addition, dependency on the ACE2 receptor as an entry gate has also been observed in SARS-CoV [76] and other sarbecoviruses [77,78,79]. Others have demonstrated that ACE2 decoy can effectively neutralize some sarbecoviruses [38] and continue to be effective against BA.2 sublineage of Omicron [80]. This indicates that the viral tropism of ACE2 utilization is highly conserved and thus fundamentally supports the ACE2-Fc therapeutic to serve as a reliable pan-variant intervention for COVID-19 long into the future.

## 4. Materials and Methods

### 4.1. ACE2-Fc and Neutralizing Antibodies

The DNA sequence of the extracellular domain of ACE2 (GenBank ID: NP_001358344.1) ligated to the Fc segment of human IgG1 was synthesized, cloned into pcDNA3.4 plasmid (Invitrogen), and expanded in *Escherichia coli* DH5α. Low endotoxin plasmids were prepared using PureLink HiPure Plasmid Miniprep Kit (Invitrogen, Waltham, MA, USA). The plasmid was transfected into ExpiCHO-S cells (Thermo Fisher) with ExpiFectamine CHO transfection agent (Thermo Fisher, Waltham, MA, USA) in accordance with the developer’s instructions. Cell culture was incubated at 37 °C in humidified atmosphere containing 8% CO_2_ with shaking. After 18–22 h post transfection, 6 μL ExpiFectamine CHO Enhancer was added into 1 mL of culture, and the temperature was adjusted to 32 °C. An amount of 160 μL ExpiCHO feed was added into 1 mL of culture on days 1 and 5. The cells were harvested on days 12–14. Expression medium was collected after removing the cells by centrifugation (1000× *g*, 4 °C, 10 min). The medium was centrifuged again (15,000× *g*, 4 °C, 30 min) and 0.22μm-filtered before affinity purification using HiTrap MabSelect SuRe (Cytiva, Marlborough, MA, USA) in AKTA avant (Cytiva). Proteins were eluted with five column volumes (CV) of 100 mM sodium citrate (pH 3.0). The pH of the eluates was adjusted to 7.2 by adding 1 M Tris base. The protein solution was then exchanged with phosphate-buffered saline (PBS), concentrated by centrifugal filtration, and passed through a 0.22 μm filter to generate the ACE2-Fc product. This product was aliquoted and placed into a -80 °C freezer before usage. The concentrations of ACE2-Fc product were determined based on optical density (OD) 280 nm measurement with the molar extinction coefficients of ACE2-Fc protein. For long-term storage, ACE2-Fc was stored in a formulation buffer containing phosphate-buffered saline, 9% trehalose, and 0.01% polysorbate 80.

Monoclonal antibodies 13A12, 8G9, and 10D4 were obtained previously as mentioned in our other report [28]. Briefly, peripheral blood mononuclear cells from COVID-19 convalescents were collected to enrich B cells. Single memory B cells that secrete anti- RBD antibody were identified by staining with fluorescent antibodies. 7AAD−, CD19+, CD27+, IgG+, and RBD+ were simultaneously gated to sort out the target cell population using fluorescence-activated cell sorting (FACS). The sorted cells were suspended with lysis solution and then stored in −80 °C freezer. Thereafter, a reverse transcription polymerase chain reaction (RT-PCR) was performed to convert B cell mRNA into cDNA, and then a PCR was performed to rescue the V genes. V genes were subsequently subjected to Sanger sequencing. Finally, the VH and VL sequences were codon-optimized and synthesized. A pcDNA3.4 backbone was used to incorporate these genes for transient expression using ExpiCHO expression system. The expression and subsequent purification processes were the same as those for ACE2-Fc. A formulation buffer (10mM histidine-HCl (pH 5.5), 9% trehalose, and 0.01% polysorbate 80) was adopted to formulate the antibodies for long-term stability.

### 4.2. Cells

HEK-293T cells stably expressing ACE2 receptor (ACE2-293T) were constructed using lentiviral transduction method [28]. Briefly, a lentiviral system harboring the sequence of the ACE2 receptor gene (GenBank ID: NP_001358344.1) was transduced into HEK-293T cells (ATCC) to obtain the ACE2-293T cell pool. This cell pool was selected with 10 μg/mL puromycin (Beyotime Biotechnology) for 1 week. Transduction efficiency was determined by flow cytometry using a primary antibody of S1-mFc recombinant protein (Sino Biological, Beijing, China) and a secondary antibody of FITC conjugated AffiniPure Goat Anti-Mouse IgG (Jackson ImmunoResearch, Chester County, PA, USA). The cell pool was then subjected to fluorescence-activated cell sorting using a FACS Aria III instrument (BD) to sort the cell population with the top 1% fluorescence intensity. The cell population was then retained and expanded for subsequent use. ACE2-293T cells were grown in Dulbecco’s modified Eagle’s medium (DMEM) with high glucose (4500 mg/L), 10% fetal bovine serum (FBS), 100 units/mL penicillin and streptomycin at the temperature of 37 °Cand atmosphere of 5% CO_2_. ExpiCHO Expression Medium (Thermo Fisher) was used to culture ExpiCHO-S cells (Thermo Fisher) at 37 °C, 8% CO_2_, 125 rpm. DMEM supplemented with 10% FBS was used to culture Vero E6 (ATCC, CRL-1586). BHK-21 cells (ATCC, CCL-10) were grown in DMEM with 10% FBS, 100 units/mL penicillin, and 100 μg/mL streptomycin at 37 °C, with 5% CO_2_.

### 4.3. HIV-Based Pseudovirus Package and Neutralization

For VOCs/VOIs package and neutralization, the amino acid sequences of VOCs/VOIs spike protein with truncation of the last 21 amino acids in the C-terminal [81] were codon-optimized and synthesized (General Biological, Chuzhou, Anhui, China). These sequences were then cloned into the pMD2.G backbone by substituting the G glycoprotein of the vesicular stomatitis virus (VSV-G). When HEK-293T cells were grown at ~80% confluence in a 10 cm dish, they were co-transfected with plasmid pLVX-Luc2 (12 μg) that encodes luciferase reporter genes, plasmid psPAX2 (8 μg) that encodes gag and pol protein, and plasmid pMD2G-ΔG-SpikeΔ21 (4 μg) that encodes the spike protein of VOCs/VOIs using transfection reagent of Lipofectamine 3000 (Invitrogen). After 12 h, fresh DMEM medium with 10% FBS (Gibco) was added in and then incubated for another 48 h. The viral medium was harvested and centrifuged at 12,000× *g* for 3 min to eliminate cell debris. ACE2-293T cells were distributed in a white 96-well tissue culture plate (Corning, Corning, NY, USA) (50 μL/well, 1 × 10^4^ cells/well) the day before. The next day, 5 μL of harvested viral supernatant (~10,000 relative luminescence units (RLU)) was diluted with medium (90% DMEM + 10% FBS) to 50 μL volume and then incubated with equal volume of ACE2-Fc (10-fold serially diluted from 100 μg/mL, 9 dilutions). Thus, the final starting dilution was 50 μg/mL for ACE2-Fc. The mixture was incubated at 37 °C for 30 min to neutralize the virus. Blank control with equal amounts of pseudovirus but without ACE2-Fc was set. The pseudovirus/ACE2-Fc mixture was then added to the ACE2-293T cells. All operations were conducted in a biosafety level 2 (BSL-2) laboratory at Shanghai Jiao Tong University. After an additional 48 h of incubation, the degree of viral entry was determined by luminescence using the ONE-Glo™ Luciferase Assay System (Promega, Madison, WI, USA) in an Infinite M200 Pro microplate reader (TECAN). RLU values were normalized based on the values of blank control wells. Dose- neutralization-rate curves were fitted into a four-parameter nonlinear regression equation using GraphPad Prism 8 software (GraphPad Software, San Diego, CA, USA).

For the single-residue mutated variants package and neutralization, site-directed mutagenesis was conducted based on the sequence of the wild-type spike (GenBank ID: YP_009724390.1) to construct these single-residue mutation sequences. The lentiviral package and neutralization experimental procedures were the same as that of VOCs/VOIs package and neutralization.

### 4.4. Replication-Competent VSV-SARS-CoV-2-S Virus Model Recovery

The recovery of the infectious VSV-SARS-CoV-2-S virus was performed using the plasmid-based rescue method [82]. Briefly, a plasmid that encodes the VSV genome sequence was changed to delete the native G glycoprotein and then incorporate the SARS-CoV-2 wild-type S glycoprotein. This VSV genome sequence also incorporates a GFP reporter gene and was designated as pVSV-ΔG-SpikeΔ21 plasmid. A recombinant vaccinia virus that encodes T7 RNA polymerase was used to infect BHK-21 cells, and the cells were simultaneously transfected with the pVSV-ΔG-SpikeΔ21 plasmid and the plasmids that encode VSV L G, P, and N proteins using polyethylenimine (PEI) (Polysciences, Warrington, PA, USA). On days 2–7 post-transfection, the vaccinia virus was removed by filtering through a 0.22-µm filter, and then the supernatant was added into fresh BHK-21 cells daily, until the appearance of a typical cytopathic effect (CPE). A pCAG-VSV-G plasmid that encodes VSV-G protein was used to transfect BHK-21 cells to expand the rescued virus. Thereafter, the rescued virus was added into fresh Vero E6 cells without complementing VSV-G protein. Viral supernatants were aliquoted and then placed into a -80 °C freezer. The viral stocks were expanded from Vero E6 cells. A plaque assay was performed on Vero E6 cells to determine the titer of the virus. The generation of VSV-SARS-CoV-2-S and its use in tissue culture were carried out at the BSL-2 lab at Shanghai Jiao Tong University. Before application in the virus evolution experiment, the virus population was passaged seven times to generate sequence diversity.

### 4.5. Virus Evolution Simulation Assay

Vero E6 cells were distributed into a 96-well plate (1.25 × 10^4^ cells/well) overnight. The following day, replication-competent VSV-SARS-CoV-2-S viral population at a multiplicity of infection (MOI) of 0.01 in 50 μL medium was mixed with equal volume of ACE2-Fc (5-fold serially diluted from 100 μg/mL, 11 serial dilutions) to neutralize susceptible variants. Thus, the final starting dilution was 50 μg/mL for ACE2-Fc. The ACE2-Fc/virus mixture was incubated at 25 °C for 30 min and then added into the Vero E6 cells. After incubation for 72 h, the cells were imaged, and the wells showing evidence of viral replication (≥20% GFP-positive cells) were recorded. Among the recorded wells (containing serial concentrations of ACE2-Fc), the well containing the suboptimal concentration of ACE2-Fc was chosen for passage. Five microliters of the supernatant from this well were harvested. The supernatant (5 μL) was diluted with the medium to a total volume of 50 μL and incubated with serially diluted ACE2-Fc. The mixture was then used to infect fresh Vero E6 cells in 96-well plates as previously described. This procedure was repeated 15 rounds. The workflow of this assay is shown in Figure S4 with biorender.com (accessed on 2 April 2022).

To reveal the mutations in antibody-escaped viral populations or p15 viral populations, viral supernatants were harvested and then centrifuged at 1000× *g*, 3 min, 4 °C. A second centrifugation of 15,000× *g*, 3 min, 4 °C was then conducted. The clarified supernatant containing the viral populations was subjected to whole viral genome high-throughput RNA sequencing (Tpbio).

### 4.6. Escaped Viral Population Neutralization

The escaped viral populations 13A12, 8G9, and 10D4 were harvested to evaluate ACE2-Fc neutralization. For titration of ACE2-Fc, a 100 μg/mL initial dilution was 5-fold serially diluted over nine dilutions and a 2.5 μL aliquot of each escaped viral population was mixed with 52.5 μL of the medium to obtain a total volume of 55 μL. Then, a 55 μL aliquot of viral solution was mixed with an equal volume aliquot of serially diluted ACE2-Fc for 30 min at 25 °C in a 96-well plate. After that, 100 μL of the mixture was transferred to Vero E6 cells plated at 1.25 × 10^4^ cells/well in 50 μL medium in 96-well plates one day before. Therefore, the final initial dilution was 50 μg/mL for ACE2-Fc. The cells were then cultured for 36 h and trypsinized with TrypLE Express (Thermo Fisher), followed by quantification of GFP fluorescence intensity using a CytoFLEX Flow Cytometer (Beckman Coulter, Brea, CA, USA). Mean fluorescence intensity (MFI) was normalized based on the MFI value derived from cells infected with the escaped viral population without ACE2-Fc. A four-parameter nonlinear regression analysis was performed to calculate the half-maximal inhibitory concentration (IC_50_) of ACE2-Fc using GraphPad Prism 8 software.

### 4.7. Structural Analysis Based on Atomic Model

The high-resolution RBD-ACE2 complex structure model published in Science [11] was downloaded from the Protein Data Bank (PDB) (accession numbers:6M17, 2.90 Å). To study the RBD residues within a 4 Å distance from ACE2, a calculation criterion, select zone parameter <4 Å from the entire ACE2 molecule, was applied to the model. This visualization was repeated with two other high-resolution RBD-ACE2 complex structures published in Nature [8,9] (PDB ID:6M0J and 6VW1) and validated quantities of interaction between ACE2 and RBD.

For N487T mutated structural analysis, a smoothed backbone-dependent rotamer library was used to predict the T487 structure. The protein side-chain rotamer library developed by Dunbrack et al. was used extensively for structure prediction [39]. This library takes advantage of a large dataset of experimental data and enables a search space for conformations to derive accurate and smooth density estimates of rotamer populations and their relative frequencies. The most probable T487 rotamer structure was selected from the resulting rotamer populations. These analyses were performed using UCSF Chimera software [83].

## Data Availability

The high-throughput sequencing data presented in this study are openly available in Sequence Read Archive (SRA) database, reference number: SRR17551408–SRR17551415.

## Supplementary Materials

The following supporting information can be downloaded at: https://www.mdpi.com/article/10.3390/ph15081002/s1, Figures S1–S6 were provided as Supplementary Materials.
