# Supplementary material for: Recombinant Decoy Exhibits Broad Protection against Omicron and Resistance Potential to Future Variants
Source: Pharmaceuticals (Basel). 2022 Aug 15;15(8):1002. doi: 10.3390/ph15081002 (PMC9413901; doi:10.3390/ph15081002)
Supplement: Supplementary file 1 [file pharmaceuticals-15-01002-s001.zip › pharmaceuticals-1826175-supplementary.pdf]

VOC

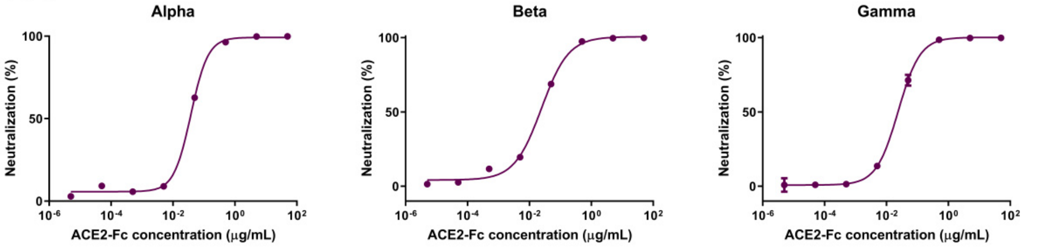

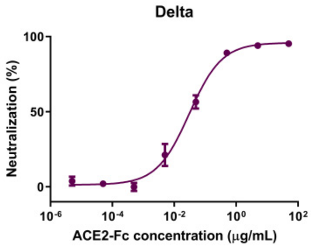

	IC_{50} (μg/mL)	95% CI of IC_{50} (μg/mL)	DF	R squared
Alpha	0.03784	0.03342 to 0.04284	12	0.9981
Beta	0.02455	0.01980 to 0.03044	12	0.9965
Gamma	0.02394	0.02023 to 0.02833	12	0.9976
Delta	0.03059	0.02045 to 0.04576	12	0.9899

VOI

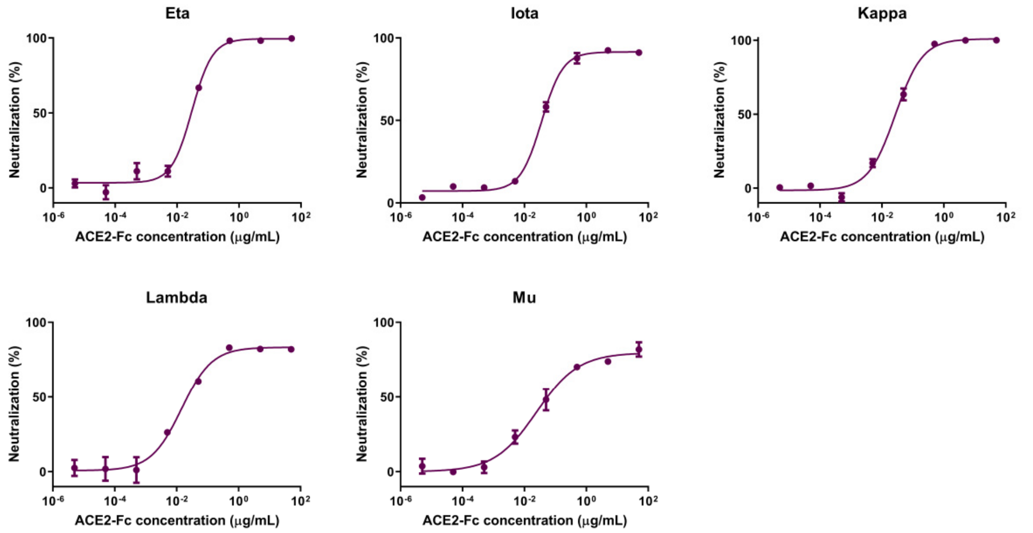

SARS-CoV

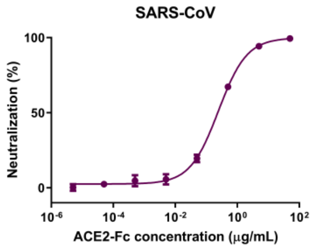

	IC_{50} (μg/mL)	95% CI of IC_{50} (μg/mL)	DF	R squared
Eta	0.03020	0.02157 to 0.04227	12	0.9891
Iota	0.03603	0.02948 to 0.04403	12	0.9956
Kappa	0.02727	0.02046 to 0.03635	12	0.9937
Lambda	0.01440	0.008238 to 0.02517	12	0.9801
Mu	0.02466	0.01213 to 0.05016	12	0.9784
SARS-CoV	0.2466	0.1968 to 0.3090	12	0.9962

Figure S1. ACE2-Fc efficiently neutralized VOCs/VOIs as well as SARS-CoV-1. Lentiviral particles pseudotyped with S protein of VOCs/VOIs or SARS-CoV were used to evaluate their susceptibility to ACE2-Fc neutralization. Neutralization ratios were calculated as described in Figure 1 (mean±SD, N=2). IC_{50} values were calculated by four parameter nonlinear regression fitting.

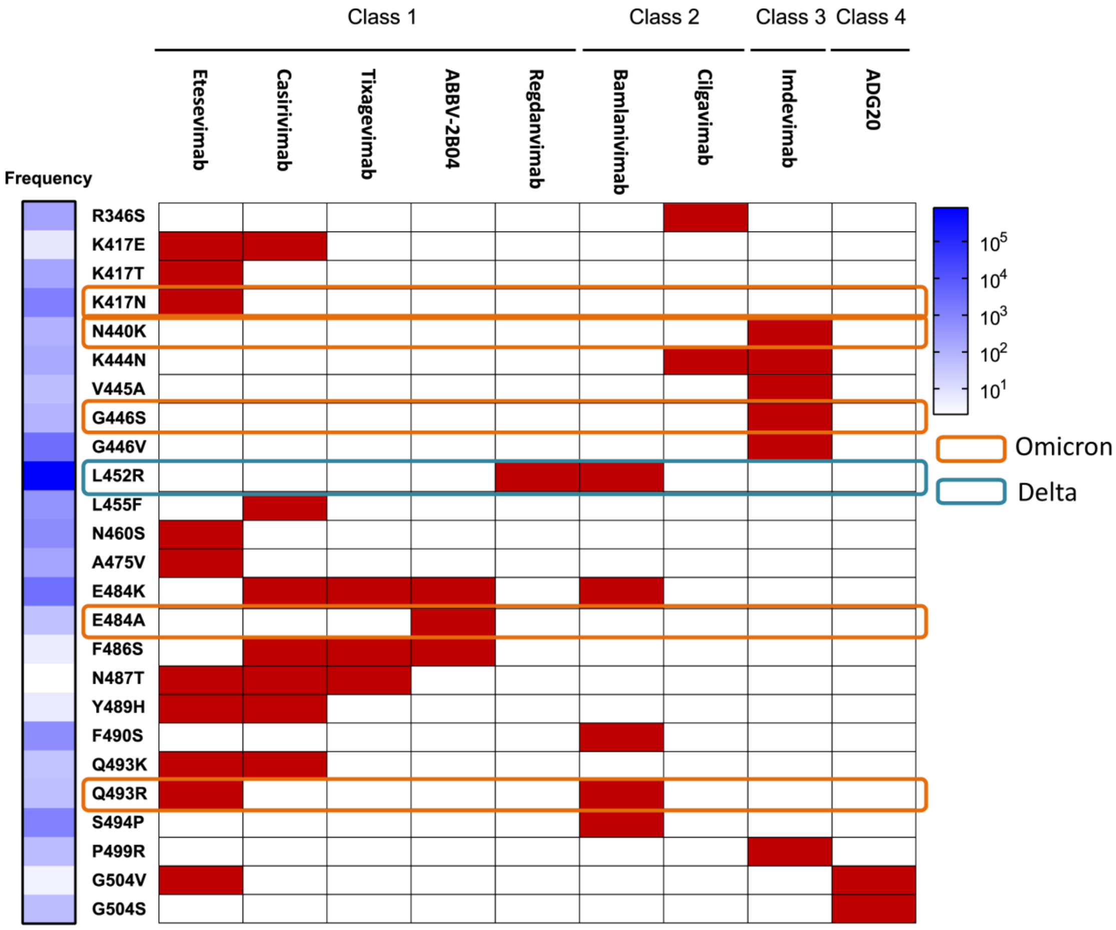

Figure S2. Summary of single amino-acid mutations that were selected upon the selective pressure of antibody therapeutics either EUA authorized or in clinical trials. Red boxes indicate escape. Escape information of each antibody were obtained from these references: Etesevimab [1], Casirivimab [1-4], Tixagevimab [5], ABBV-2B04 [6], Regdanvimab [7], Bamlanivimab [3], Cilgavimab [5], Imdevimab [1,2], and ADG20 [3].

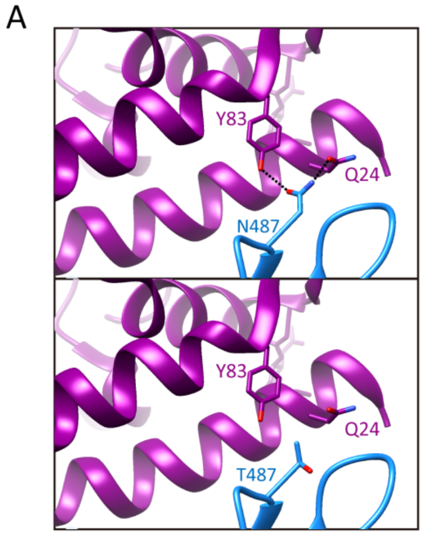

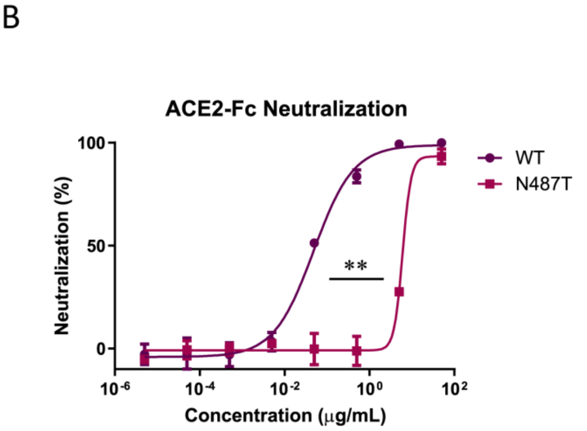

	P value	DF	F (DFn, DFd)
WT vs. N487 using two-way ANOVA	0.0026	1	F (1, 2) = 377.8

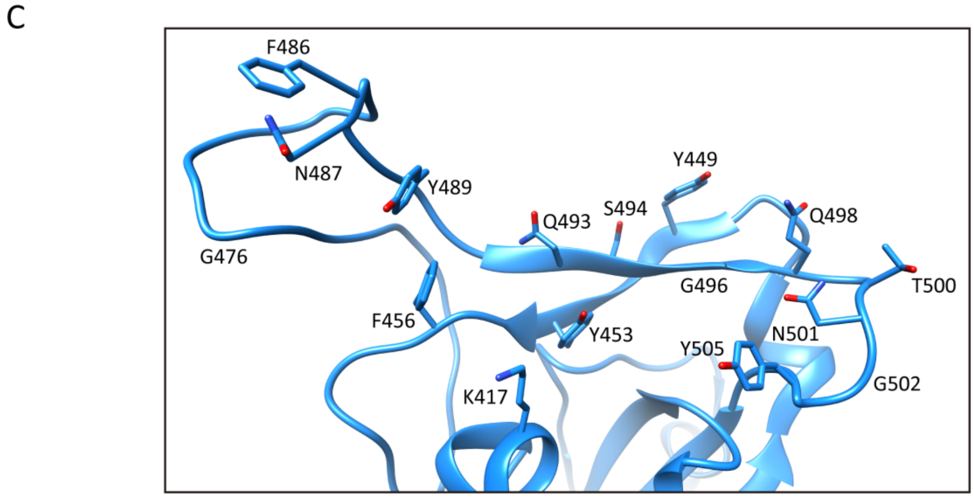

Figure S3. Regardless of sensitive to mutations on N487, high concentration of ACE2-Fc neutralized N487T variant. (A) Structural analysis based on published high-resolution ACE2-RBD complex structure model (see Materials and Methods) showed there are two hydrogen bonds formed between N487 of RBD and Y83 or Q24 of ACE2. However, T487 lost interaction with ACE2. **(B)** ACE2-Fc neutralized N487T variant. Neutralization ratios are displayed as mean±SD, N=2. Comparison was carried out using two-way ANOVA statistical analysis. **$p<0.01$. **(C)** ACE2-RBD interface has a large attachment area and quantities of interacting residues. Residues on RBD within 4 Å distance from ACE2 were analyzed.

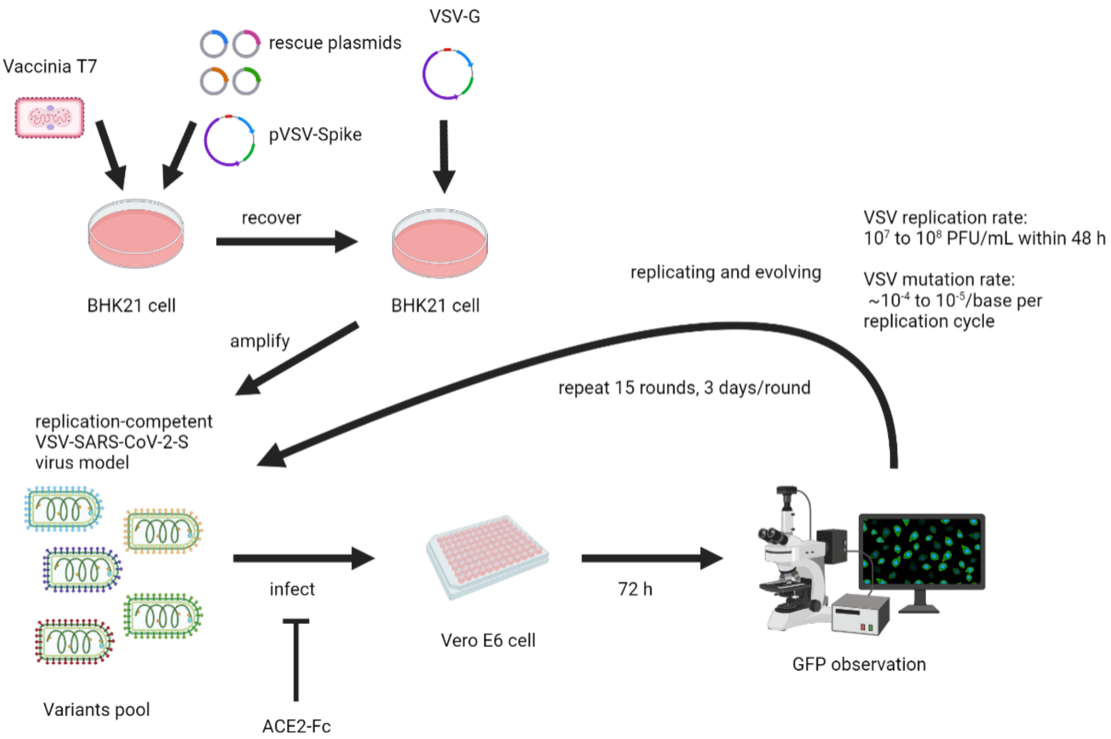

Figure S4. Workflow of generation of replication-competent VSV-SARS-CoV-2-S virus model and subsequent evolution experiment. See Materials and Methods part for detailed methodology description.

A

Position	74	79	247	417	475	486	682	685	685	687	687	754	808	813	982	1081	1181
Ref Residue	N	F	S	K	A	F	R	R	R	V	V	L	D	S	S	I	K
Alt Residue	K	L	R	E	V	L	G	S	H	G	M	R	G	L	N	V	T
Inoculum	61.25%	63.24%	52.82%	0%	0%	0%	0%	43.85%	55.01%	0%	0%	0%	5.19%	38.57%	46.13%	0%	0%
13A12	100.00%	99.94%	0%	100.00%	14.72%	6.22%	0%	0%	99.97%	0%	0%	0%	0%	100.00%	0%	0%	0%
13A12+ACE2	50.53%	52.66%	0%	13.61%	0%	0%	45.28%	0%	55.92%	8.71%	5.02%	19.98%	0%	44.31%	0%	21.69%	5.37%

B

Position	74	79	247	354	389	484	685	685	808	813	982
Ref Residue	N	F	S	N	D	E	R	R	D	S	S
Alt Residue	K	L	R	D	N	K	S	H	G	L	N
Inoculum	61.25%	63.24%	52.82%	0%	0%	0%	43.85%	55.01%	5.19%	38.57%	46.13%
8G9	99.99%	100.00%	0%	99.92%	100.00%	98.89%	0%	99.93%	0%	0%	0%
8G9-ACE2	100.00%	99.96%	0%	0%	0%	0%	0%	100.00%	5.82%	0%	0%

Frequency <10%	Frequency 10%<x<50%	Frequency >50%

C

Position	74	79	87	247	468	568	685	685	792	808	813	935	982
Ref Residue	N	F	N	S	I	D	R	R	P	D	S	Q	S
Alt Residue	K	L	Y	R	T	N	S	H	T	G	L	R	N
Inoculum	61.25%	63.24%	0%	52.82%	0%	0%	43.85%	55.01%	0%	5.19%	38.57%	0%	46.13%
10D4	100.00%	100.00%	100.00%	0%	100.00%	95.27%	0%	99.88%	0%	6.68%	0%	0%	0%
10D4-ACE2	100.00%	100.00%	0%	0%	0%	0%	0%	0%	10.22%	6.66%	0%	9.05%	0%

Figure S5. Mutations on the spike proteins of escaped viral population and passages 15th viral population of cocktails. (A) 13A12, (B) 8G9, and (C) 10D4 escaped viral population and p15 viral populations of corresponding cocktails were subjected to high-throughput sequencing. The RBD region is highlighted in blue.

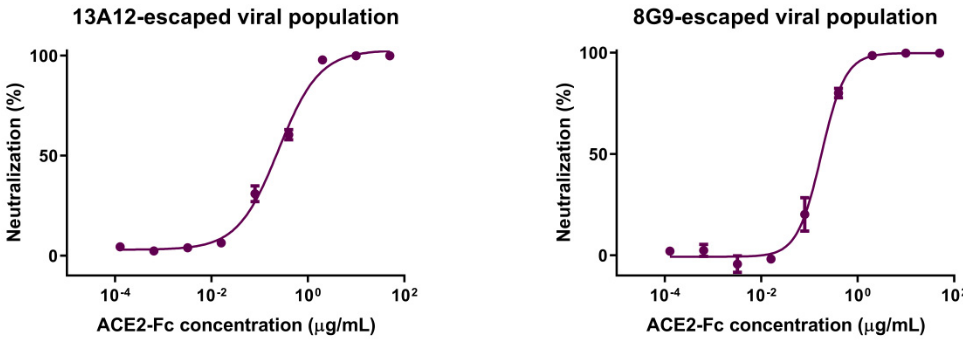

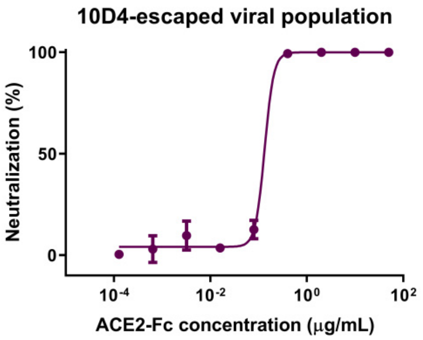

	IC_{50} (μg/mL)	95% CI of IC_{50} (μg/mL)	DF	R squared
13A12-escaped	0.2455	0.1922 to 0.3136	14	0.9930
8G9-escaped	0.1759	0.1434 to 0.2158	14	0.9925
10D4-escaped	0.1331	0.05020 to 0.3529	14	0.9917

Figure S6. ACE2-Fc efficiently neutralized 13A12-, 8G9-, and 10D4-escaped viral populations. Escaped viral populations were harvested and neutralized using serially diluted ACE2-Fc, following by applying to Vero E6 cells to measure the GFP fluorescence by flow cytometry. Neutralization ratios were calculated based on the MFI values (mean±SD, N=2). IC_{50} values were calculated by four parameter nonlinear regression fitting.

References for Supplementary Materials:

1. Starr, T.N.; Greaney, A.J.; Addetia, A.; Hannon, W.W.; Choudhary, M.C.; Dingens, A.S.; Li, J.Z.; Bloom, J.D. Prospective mapping of viral mutations that escape antibodies used to treat COVID-19. *Science* **2021**, *371*, 850-854, doi:10.1126/science.abf9302.
2. Baum, A.; Fulton, B.O.; Wloga, E.; Copin, R.; Pascal, K.E.; Russo, V.; Giordano, S.; Lanza, K.; Negron, N.; Ni, M.; et al. Antibody cocktail to SARS-CoV-2 spike protein prevents rapid mutational escape seen with individual antibodies. *Science* **2020**, *369*, 1014-1018, doi:10.1126/science.abd0831.
3. Rappazzo, C.G.; Tse, L.V.; Kaku, C.I.; Wrapp, D.; Sakharkar, M.; Huang, D.; Deveau, L.M.; Yockachonis, T.J.; Herbert, A.S.; Battles, M.B.; et al. Broad and potent activity against SARS-like viruses by an engineered human monoclonal antibody. *Science* **2021**, *371*, 823-829, doi:10.1126/science.abf4830.
4. Greaney, A.J.; Loes, A.N.; Crawford, K.H.D.; Starr, T.N.; Malone, K.D.; Chu, H.Y.; Bloom, J.D. Comprehensive mapping of mutations in the SARS-CoV-2 receptor-binding domain that affect recognition by polyclonal human plasma antibodies. *Cell Host Microbe* **2021**, *29*, 463-476.e466, doi:10.1016/j.chom.2021.02.003.
5. Dong, J.; Zost, S.J.; Greaney, A.J.; Starr, T.N.; Dingens, A.S.; Chen, E.C.; Chen, R.E.; Case, J.B.; Sutton, R.E.; Gilchuk, P.; et al. Genetic and structural basis for SARS-CoV-2 variant neutralization by a two-antibody cocktail. *Nat Microbiol* **2021**, *6*, 1233-1244, doi:10.1038/s41564-021-00972-2.
6. Liu, Z.; VanBlargan, L.A.; Bloyet, L.M.; Rothlauf, P.W.; Chen, R.E.; Stumpf, S.; Zhao, H.; Errico,

J.M.; Theel, E.S.; Liebeskind, M.J.; et al. Identification of SARS-CoV-2 spike mutations that attenuate monoclonal and serum antibody neutralization. *Cell Host Microbe* **2021**, *29*, 477-488.e474, doi:10.1016/j.chom.2021.01.014.

7. McCallum, M.; Bassi, J.; De Marco, A.; Chen, A.; Walls, A.C.; Di Iulio, J.; Tortorici, M.A.; Navarro, M.J.; Silacci-Fregni, C.; Saliba, C.; et al. SARS-CoV-2 immune evasion by the B.1.427/B.1.429 variant of concern. *Science* **2021**, *373*, 648-654, doi:10.1126/science.abi7994.
